# Enter the dragon – China's journey to the hearing world

**DOI:** 10.1179/1467010013Z.00000000080

**Published:** 2013-03

**Authors:** Qi Liang, Brendan Mason

**Affiliations:** Cochlear Limited, China

**Keywords:** Cochlear implant, China infrastructure, Insurance coverage, Training, Hearing outcomes

## Abstract

**Context:**

China's population of 1.3 billion represents nearly 20% of the world's population. The current live birth rate in China is 17 million per year, compared with 4.1 million in the USA in 2009. Ministry of Health figures from China identify 115 000 children under the age of 7 years with severe-to-profound deafness and 30 000 babies born each year with hearing impairment.

**Newborn screening:**

Universal Newborn Hearing Screening (UNHS) has been implemented in China since 1999. By 2010 UNHS was implemented in 20 of the 32 Chinese provinces. In large cities 95% of babies are screened in hospital-based programs. In more remote areas babies with high-risk factors for hearing loss are referred to screening centers within 1 month of birth and leaflets about identifying deafness are distributed.

**Cochlear implants:**

Since 1995 more than 10 000 people in China have received cochlear implants (CIs) and 85% of these implant recipients have been children under the age of 7 years.

**Financing of CIs:**

China is in the process of developing a national reimbursement scheme for medical care. The first multichannel implant was performed in 1995. In 2005, a private financier provided more than 1500 implants for children under the age of 5 years. In 2009, the Chinese government set up a project to implant 1500 children aged 1–5 years over the next 3 years, with provision of the equivalent of US$65.4 million to pay for the devices, surgery, mapping, and rehabilitation. By 2011, the government had agreed to fund implants for an additional 17 000 children over 4 years.

**Training of professionals:**

Schemes have been developed to train surgeons, Audiologists, and those involved in rehabilitation of implanted children in China.

**Outcome assessment:**

Standardized outcome tests are being developed for CI recipients. There are two large-scale ongoing outcome studies in progress. CI penetration in China is currently less than 5% of potential pediatric candidates, but cochlear implantation is continuing to expand at great speed, and it is hoped that the infrastructure and capacity will continue to grow and develop.

## Introduction

The dragon is an imaginary and mysterious creature that ancient Chinese people feared and worshiped. It is said that the dragon senses sound through its horns because it does not have ears. Chinese people often call themselves descendants of the dragon. Interestingly, the Mandarin Chinese pronunciation for ‘Deaf’ and ‘Dragon’ are the same. Written in Chinese, deaf [聾] is composed of two Chinese characters [龍], which mean a dragon, and [耳], an ear. These similarities in the dragon's mythical anatomy, verbal pronunciation, and written character culturally connect the Dragon with people who are deaf in China.

In Chinese culture, the dragon is a symbol of power, strength, and good luck. However, deafness remains an obstacle for millions of Chinese people. This article provides an insight into the path that Chinese people who are deaf follow to become part of the hearing world. We look at cultural attitudes and the historical development of cochlear intervention, and explore the key elements vital to ensure access to new hearing technology in the People's Republic of China.

With more than 1.3 billion people, China represents nearly 20% of the world's population (Asia Foundation, 2011). Since 1979 China's population growth has been slowed down by the one-child policy. Despite China's attempt to curb population growth, the current number of live births is about 17 million babies per year which dwarfs the US number, estimated at 4.1 million in 2009 (US Department of HHS, 2011). The size of China, its decentralized population (50% live in rural villages), and the number of births pose significant challenges for the Central Government as it grapples with current healthcare reform initiatives and the design of social insurance schemes which serve to adequately care for the biggest population in the world.

China' s economy grew at an annual rate of 9.7% from 1978 to 2006 (China Statistical Abstract, 2007). This explosive growth raised at least 210 million people from poverty. In 2007, World Health Organization ranked China's healthcare system 144th in the world out of 195 surveyed countries using GDP/capita as its measure (World Health Organization, 2000). In 2007, China's healthcare spending amounted to less than 1% of national GDP. This situation led to the long awaited reform of the healthcare system, which included a focus on China's citizens in vulnerable situations. The China Disabled Persons Federation (CDPF) also announced a national program to assist deaf children which involves treatment with Hearing Aids and Cochlear Implants (Hu, 2009).

According to the most recent national disability survey, there were 27.8 million people with some level of hearing loss, 115 000 children under the age of 7 are affected by severe-to-profound hearing loss and 30 000 babies born each year with hearing impairment (The Ministry of Health, 2001).

**Figure 1:**
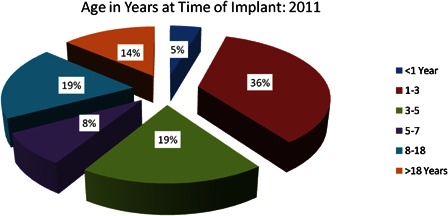
Age in years at the time of implant: 2011. Source: Cochlear Ltd (China) Database.

Since 1995, more than 10 000 people in China have received cochlear implants (CIs), a 25% average annual growth rate. Children under 7 years of age comprise 85% of the recipient population (see Fig. 1).

Today cochlear implantation has become a common intervention for deaf children because of the dedication of hearing professionals, government support, and improved affordability.

## Cultural factors

Similar to trends in industrialized countries, individuals with hearing impairment experience higher rates of unemployment, lower education levels, and are from families with lower incomes (Haualand and Allen, 2009).

Chinese Sign Language (CSL), currently used by deaf people in China, is not considered to be a discrete language by the general public. Over the past 50 years, use of CSL has not expanded to the extent that sign language has grown in other countries in part because of the stigma associated with deafness and other disabilities. In addition, Chinese people have not developed a deaf culture in the same fashion as in developed countries because of the fragmentation of services for deaf individuals (Haualand and Allen, 2009). The isolation of deaf people as well as control by the government with regard to disability services has enhanced interest in hearing technologies that allow people to live life in the mainstream. These cultural factors have helped to amplify the relatively rapid acceptance of cochlear implantation in China.

In Chinese culture, there is long tradition of families investing in their children, even in instances in which a family has few monetary resources. Parents and grandparents will do without to allow the greatest opportunity for their children. The one-child policy adds further to this focus on children's needs. Because of this, pediatrics has been the heart of CI programs in China in the past. This is now changing somewhat as attitudes towards progressive deafness in adults are recognizing both the quality of life and economic benefits of CIs for adults who have been late deafened. While most government support remains directed towards children, awareness and public education have resulted in an expansion in the number of adult CI recipients.

## Newborn screening

In 1999, the Central Government recommended that Universal Newborn Hearing Screening (UNHS) be implemented. The first phase of the program included 15 provinces chosen based on the existing infrastructure capacity and technical abilities. In 2012, hearing screening was conducted in 20 of the 32 provinces (Xingkuan, 2004).

In 2012, based on the 20 provinces undertaking screening, the incidence of hearing loss in newborn babies ranged from three to six babies per 1000 births. In large cities, more than 95% of newborns were screened using Otoacoustic emissions (OAE)/Automated Auditory brainstem response (AABR) methods. The OAE/AABR test provides a simple pass/refer result, and referrals are referred to confirmation centers where more extensive tests are conducted (Xingkuan, 2004).

**Figure 2:**
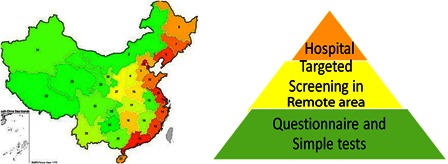
Hearing Screening in China 2012. Source: Liang & Mason (2012).

Hearing Screening in China is structured into three categories (Fig. 2):

1. *Large cities with extensive resources*: These include hospital-based UNHS programs where at least 95% of babies are screened. There are currently 20 provinces or municipalities that screen to this level.
2. *Targeted screening*: In rural and remote areas, newborns that exhibit high-risk factors are referred to screening centers within 1 month of birth.
3. *Wide dissemination of questionnaires and simple tests*: These are recommended by community doctors at a village or suburban level to monitor each child's hearing (Xingkuan, 2004).

China faces many challenges for the successful and effective implementation of its UNHS program. Historical under-spending on national healthcare combined with a large population has resulted in a massive shortage of hearing care professionals, as a result, access to hearing screening is constrained. This creates problems for discerning an effective referral pathway for newborn hearing-impaired children. Further, the lack of a national or provincial database thwarts a systematic follow- up process.

## Insurance coverage

**Figure 3:**
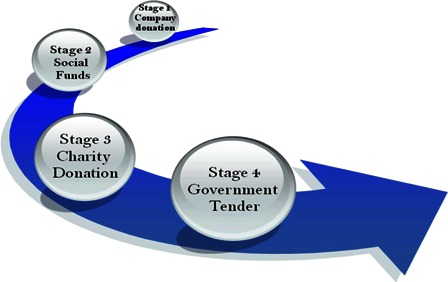
Process of funding CIs in China. Source: Cochlear China Staff.

After years of intense discussion, deliberation, and debate, in 2009 China finally unveiled its healthcare reform plan. China is in the process of developing a national reimbursement scheme for medical care. Currently, different methods of funding are being considered to ensure deaf people, especially children, are afforded access to cochlear implantation in a timely fashion after identification of hearing loss. China has gone through several stages in developing financial resources for CIs (Fig. 3).

In 1995, China's first multiple channel device was implanted. It was a donation from the CI company and was implanted at Peking Union College Hospital in Beijing. The operation generated extensive public interest in the product and the company, and a new industry was born.

In 2001, local agents formed an alliance with the Shenzhen Bank to establish a program to offer loans in order to facilitate low-cost medical services to people with hearing impairments. In the same year, the China Charity Federation and Cochlear donated a CI for a 3-year boy and government authorities from both China and Australia collaborated to launch small programs to help family with poor incomes.

In 2005, a Taiwanese philanthropist donated 200 CI systems to the Chinese government to raise awareness about CI. The following year, the same donor provided 15 000 CIs to be provided to appropriate children under the age of 5 years.

In 2009, the Chinese government announced a National Tender Project to implant 1500 children aged 1–5 years over a 3-year period. The government set aside RMB412 million (approximately US$65.4 million) to pay for the devices, the surgery, mapping, and related rehabilitation (Hu, 2009).

In 2010, several provinces in China piloted CI reimbursement programs at designated hospitals for the treatment of children with severe-to-profound deafness. Under the pilot program, 70% of the cost of CIs was reimbursed. This pilot allowed the government to test funding under a proposed national Basic Medical Insurance (BMI). The Cochlear China staff predict that full reimbursement under BMI will occur in 2016.

In 2011, the Chinese government announced that it would fund an additional 1.76 billion RMB (US$279 million) over the next 4 years to provide CIs to an additional 17 000 Chinese children. This step by the Chinese government demonstrates a strong commitment to addressing deafness, including needed reimbursement, in the country over the longer term (Hu, 2012) (see Table 1).

**Table 1:** History of cochlear implantation in China

Date	Event
Pre-2005	Private funding, user pays for CI
2005	Taiwan philanthropist pilots China Program for 200 CI systems
2006	Taiwan philanthropist donates 15 000 CI systems for deaf children
2009	China central government launches 1500 system fund pilot
2010	Provincial governments launch funding program
2011	China central government launches national funding for CI for children to consist of 17 000 systems over 4 years

## Development of China's hearing infrastructure

Success of the CI depends not only on the device itself, but also on the patient selection, surgical skills, and post-surgical rehabilitation. Infrastructure is often nascent in developing countries, and China is no exception as the hearing referral pathway that facilitates effective intervention for hearing loss is not yet fully developed (Liang, 2011). Offering training to professionals improves skills and increases the access to effective interventions like CIs. The success of any cochlear implantation program requires substantial resource expenditures. Training professionals to build China's infrastructure for cochlear implantation required considerable development and investment, particularly in the areas of surgical training, audiology, and habilitation.

### Establishing surgical training

Mao Tse Tung proclaimed in his 1930 *Red Book* ‘A single spark can start a prairie fire’. In China, the efforts to build national CI surgical skills began in 1993 when Chinese ear, nose and throat (ENT) surgeons attended training in Melbourne, Australia at the Bionic Ear Institute. The surgeons then established the first CI program after they returned to China with the first surgery completed in 1995. The process continued with senior surgeons supervising junior surgeons until they gained sufficient experience. In 2012, there were approximately 60 well-trained CI surgeons capable of independently performing the CI surgery in the Chinese mainland (Cao, 2009).

The Cochlear Implantation Surgical Certificate is another training strategy which has been developed specifically to increase skills of Chinese ENT doctors, and by the end of the certificate the surgeon is capable of independent CI surgery. The syllabus allows surgeons to learn the required CI surgical techniques by participating in online seminars, temporal bone workshops, live surgery demonstrations, and tutorial-format discussions in partnership with China's key teaching hospitals.

In addition to the surgical training programs, surgical conferences develop and expand the surgeon's existing surgical techniques and provide a platform for experienced surgeons, using their real-life experience, to share information with the developing surgeons. This in turn ensures that surgeons are both aware of the technology and also trained to safely use the technology through practical and theoretical teaching methods.

Support by the Chinese central government and the general medical community has helped the expansion of cochlear implantation in China. In 2003, the first national CI conference was held in the southern city of Changsha. Subsequently, the first Cochlear Implant Guidelines were adopted by the Chinese ENT society (Changsha, 2003). By 2007, the Ministry of Health and the Chinese Medical Association had issued Cochlear Implant Clinical Standards (The Ministry of Health, 2007).

### Audiology training

Audiology is not recognized by Chinese hospitals as a medical profession. There are only three universities which provide college-level audiology education and about 20 educational institutions which provide audiology-related training below the college level. In 1998, the first Joint Sino-Australia audiology program was started in China. Since that time, approximately 1000 audiology professionals have been trained; 65 of these professionals can now effectively program a cochlear recipient (Liang, 2011). Still, there exists a shortage of CI audiologists in China, especially in the specialized field of pediatric audiology.

In China, CI programming is often performed by non-audiologists, such as physicians, technical staff, nurses, or rehab teachers. In 2007, the Cochlear Training and Education Centre (CTEC) was established to improve the technical skills of these Chinese professionals. CTEC Audiology provides a central base for audiologists across China with a particular focus on best practice pediatric CI programming. To date, 105 professionals have utilized the resources of CTEC to improve their technical skills and develop their ability to respond proactively to the healthcare needs of China's population with hearing loss. It is important to note that CTEC does not intend to replace formal audiology education. Rather, CTEC enhances on-the-job skill requirements in the form of vocational training.

In 2010, the Cochlear Practical Audiology Diploma (C-PAD) was developed to address the growing disparity between the number of qualified mapping audiologists and professionals need to provide services to the rapidly increasing number of CI recipients. The program offers a specially designed curriculum consisting of theory and practical mapping instruction. It covers three modules taught as three individual certificates: (1) Introductory Audiology, (2) Interventions in Audiology, and (3) Implementing Audiology. The duration of the course is typically 6 months and the core coursework modules are delivered by distance learning using an online platform known as the Cochlear Virtual University. So far, 130 students are working their way towards the C-PAD qualification. In June 2011, the first cohort of eight students graduated and received the C-PAD certificate (Liang, 2011).

### Rehabilitation training

In China there is no formal university accredited program to train teachers for the deaf or speech pathologists. Typically, intending practitioners start with a general degree in special needs education then go on to undertake a certificate in their desired specialization. The proportion of rehabilitation professionals with a university degree is low. Historically, the education of deaf children in China has been delivered through residential or day-care learning. Education of the deaf has focused on the development of oral language skills taught primarily through lip-reading.

The China Rehabilitation Research Center for Deaf Children (CRRCDC) has addressed this issue, and now offers nationwide instruction and management in the rehabilitation fields in the form of vocational on-the-job training. A network of rehabilitation for hearing-impaired children has been set up across China, including 33 provincial, 612 city-level, and 926 country/village/community-level rehabilitation centers. According to the CRRCDC report, there are 2879 teachers in government rehabilitation centers and 1100 teachers in private rehabilitation centers across China (Sun, 2009).

In 2007, the CRRCDC collaborated with the Royal Institute for Deaf and Blind Children, Renwick Centre, Australia, to develop a 7-month ‘Auditory Verbal Theory and Practice’ teacher training course, which provides an introduction to auditory verbal theory in China. Other programs offered include short courses in teacher training on specific topics as well as an early intervention Auditory Verbal Therapy program (Kendrick, 2008).

In 2009, the CRRCDC signed a 5-year agreement with the Children's Hearing Foundation (CHF) in Taiwan for the provision of Auditory Verbal Therapy (AVT) teaching in China. Special 3-month intensive programs were developed to improve the skills of rehabilitation teachers in China.

While the development of these programs faced significant challenges, they can be considered important steps in rehabilitation training in China. These programs contribute significantly to building rehabilitation infrastructure in China and improving the quality of life for the 27.8 million Chinese people living with hearing loss.

## Recipient outcomes

It is generally accepted that recipient outcomes are the most important measure of a CI program. However, objective tests that document outcomes remain scarce in China. Without standardized objective tests, the prescription of effective corrective actions cannot be implemented. China is in the early stages of responding to this challenge. The development of assessment tools opens a new dimension on outcomes research for CI children recipients in China, for example the Mandarin Early Speech Perception Test, a pediatric test battery with a structure paralleling the English ESP, and Mandarin Pediatric Lexical Tone and Disyllabic Word Picture Identification Test in Noise (Zheng *et al*., 2010).

There are currently two large-scale ongoing outcomes studies on CI outcomes in China:

1. *A multi-center pediatric longitudinal study*. Four CI clinics are investigating the effect of age of implantation on auditory perception and speech recognition performance for Mandarin-speaking CI children. Comparisons are made between the auditory perception and speech recognition performance of CI children and the normal hearing population.
2. A joint study with the House Ear Institute in the USA and West China Hospital in Chengdu, China. This 4-year study will evaluate the development of speech and language abilities in 120 children who received a CI before the age of 5 years. This research will identify key factors in the early development of speech and language abilities of normal hearing children and compare such factors to those observed in profoundly hearing-impaired children who use CIs and/or hearing aids (House Ear Institute, 2009).

The preliminary data from the studies indicate that the performance of Mandarin-speaking CI children is comparable to that of US children with CIs. Although the results of studies on CI outcomes of Mandarin-speaking children showed variable outcomes, it is evident that the majority of children benefited from CI intervention.

## Summary

A successful program for CIs in China requires raising the awareness of CIs and hearing impairment in general. When multi-channel CIs entered the Chinese market in 1995, there were no newborn hearing testing programs or trained audiology professionals, no funding available for cochlear implantation and limited numbers of physicians with CI surgical experience. Building infrastructure, securing financial resources, improving outcomes of cochlear implantation, and educating surgeons, health professionals, and governmental officials have been the main challenges for the CI industry in China.

Today, CI penetration is less than 5% of the pediatric candidate population. It is estimated that 19 000 children are born with a severe-to-profound hearing loss annually and many would benefit from cochlear implantation. Cochlear implantation is expanding in China at an amazing speed. It is hoped that with enhanced infrastructure and capacity, China will be able to address the needs of its hearing-impaired community and improve the quality of life of children and adults across this diverse and dynamic nation.
